# Increased Plasma DPP4 Activity Is an Independent Predictor of the Onset of Metabolic Syndrome in Chinese over 4 Years: Result from the China National Diabetes and Metabolic Disorders Study

**DOI:** 10.1371/journal.pone.0092222

**Published:** 2014-03-19

**Authors:** Fan Yang, Tianpeng Zheng, Yun Gao, Attit Baskota, Tao Chen, Xingwu Ran, Haoming Tian

**Affiliations:** 1 Department of Endocrinology and Metabolism, West China Hospital of Sichuan University, Sichuan, P. R. China; 2 Department of Endocrinology and Metabolism, Affiliated Hospital of Guilin Medical University, Guangxi, P. R. China; University of Modena and Reggio Emilia, Italy

## Abstract

**Aims:**

To determine whether fasting plasma Dipeptidyl Peptidase 4 (DPP4) activity and active Glucagon-Like Peptide-1 (GLP-1) were predictive of the onset of metabolic syndrome.

**Methods:**

A prospective cohort study was conducted of 2042 adults (863 men and 1,179 women) aged 18-70 years without metabolic syndrome examined in 2007(baseline) and 2011(follow-up). Baseline plasma DPP4 activity was determined as the rate of cleavage of 7-amino-4- methylcoumarin (AMC) from the synthetic substrate H-glycyl-prolyl-AMC and active GLP-1 was determined by enzymoimmunoassay.

**Results:**

During an average of 4 years of follow-up, 131 men (15.2%) and 174 women (14.8%) developed metabolic syndrome. In multiple linear regression analysis, baseline DPP4 activity was an independent predictor of an increase in insulin resistance over a 4-year period (P<0.01). In multivariable-adjusted models, the odds ratio (OR) for incident metabolic syndrome comparing the highest with the lowest quartiles of DPP4 activity and active GLP-1 were 2.82, 0.45 for men and 2.48, 0.36 for women respectively. Furthermore, plasma DPP4 activity significantly improved the area under the ROC curve for predicting new-onset metabolic syndrome based on information from metabolic syndrome components (Both P<0.01).

**Conclusions:**

DPP4 activity is an important predictor of the onset of insulin resistance and metabolic syndrome in apparently healthy Chinese men and women. This finding may have important implications for understanding the aetiology of metabolic syndrome.

**Trial Registration:**

#TR-CCH-Chi CTR-CCH-00000361

## Introduction

The metabolic syndrome is characterized by abnormal glucose tolerance, elevated blood pressure, hypertriglyceridemia, low HDL cholesterol, central obesity, microalbuminuria and insulin resistance, subjects with metabolic syndrome are at increased risk for type 2 diabetes and cardiovascular disease [1]–[2].Given the high prevalence of the metabolic syndrome and its severe consequences, it is essential to understand its biomarkers in population-based longitudinal studies. It is well established that central obesity is the hallmark of the metabolic syndrome [3]. A complex cross-talk scenario between adipose tissue and other organs has been found to underlie the progression of the metabolic syndrome [4]. This is mainly attributed to the huge number of adipokines which are proteins and peptides released by various adipose tissue cells. Enlargement of adipose tissue leads to dysregulation of adipokine secretion, representing major link between obesity and metabolic syndrome. Since the metabolic syndrome is closely linked to obesity and adipose tissue dysfunction, adipokines are strong candidates to predict the development of metabolic syndrome.

Dipeptidyl peptidase-4 (DPP4) or T-cell activation antigen CD26 (EC 3.4.14.5.) is a serine exopeptidase belonging to the S9B protein family that cleaves X-proline dipeptides from the N- terminus of polypeptides, such as chemokines, neuropeptides, and peptide hormones [5]. Previous studies have documented that circulating DPP4 originate from cells of the immune system and differentiated adipocytes [6]–[7]. It is found to be a novel adipokine potentially linking obesity to the metabolic syndrome [6]. Recent data suggest that the protein level of DPP4 is significantly associated with insulin resistance factors and components of metabolic syndrome [6]. However, most of the observations come from cross-sectional studies and focus on the protein level of DPP4, until recently, little is known about the ability of circulating DPP4 activity as a predictor of insulin resistance and metabolic syndrome or about its ability to predict incident metabolic syndrome beyond the information provided by each of its components among healthy individuals.

We thus studied the prospective association of plasma DPP4 activity with the risk of incident metabolic syndrome and its components, as well as the predictive value of plasma DPP4 activity in identifying in individuals who will develop incident metabolic syndrome among healthy individuals. Since DPP4 is involved in the degradation of circulating active GLP-1 to biologically inactive fragments, plasma active GLP-1 level is also studied in our research. In our study, the homeostasis model assessment of insulin resistance (HOMA-IR) was used to estimate insulin resistance.

## Methods

### Subjects

The study population was men and women, aged 18–70 years, who participated in the China National Diabetes and Metabolic Disorders Study [8], a 4-year follow-up study that aims to clarify the prevalence and development of the type2 diabetes and metabolic disorders. Subjects are volunteers who came from 3 health examination centers in Sichuan province. The Medical Research Ethics Committee of the China–Japan Friendship Hospital (Location:2 Cherry Park Street, Chaoyang District, Beijing 100029, China) reviewed and approved the present study. The written informed consent was obtained from each participant before data collection. This study was registered on the Chinese clinical trial registry (#TR-CCH-Chi CTR -CCH-00000361).

The final sample size for the present analysis was 2042 participants (863 men and 1,179 women) without metabolic syndrome at baseline. Inclusion criteria: (1) Age between 18-70 years old. (2) Long-term residing (≥5 years) in China's Sichuan province. Exclusion criteria: (1) All subjects having past history of metabolic syndrome or have been diagnosed with metabolic syndrome at baseline during screening. (2) Using varieties of drugs to control blood glucose, blood pressure, blood lipid and other drugs used in preventing complications during natural process of metabolic syndrome. (3) Subjects deprived of personal safety and presence of any of the chronic diseases including stroke, myocardial infarction, other heart, liver, renal and respiratory dysfunction were excluded as progression of these in any stage may hinder our study. (4) Pregnant subjects and subjects with malignancy. (5) Does not need assistance from the medical staffs to complete the survey done twice at baseline and during follow-up. (6) Subjects with incomplete data. The diagnostic criteria of the metabolic syndrome were based on the criteria recommended by the WHO. We used the criteria by the WHO (1999), which require presence of one of diabetes mellitus(indicated by FPG ≥7.0 mmol/L or 2 h-PG≥11.1 mmol/L), impaired glucose tolerance(IGT,indicated by 2 h-PG between 7.8–11.09 mmol/L and FPG <6.1 mmol/L), or impaired fasting glucose(IFG,indicated by FPG between 6.10–6.99 mmol/L and 2 h-PG <7.8 mmol/L),and two of the following: blood pressure≥140/90 mmHg, dyslipidemia(triglycerides[TG] >1.7 mmol/L and HDL cholesterol <0.9 mmol/L [male] or <1.0 mmol/L[female]), central obesity(waist-to-hip ratio [WHR]>0.90 [male],>0.85 [female], or BMI >30 kg/m^2^), or microalbuminuria.

### Study design

A standard questionnaire was administered by trained staff to participants to record demographic characteristics and life style risk factors [9]. Blood pressure, body weight, height, waist and hip circumference, body mass index (BMI), and waist/hip ratio (WHR) were measured and calculated using standard methods, as previously described [8]. Participants were instructed to maintain their usual physical activity and diet for at least 3 days before undergoing an oral glucose tolerance test (OGTT). After an overnight fast≥10 h, venous blood samples were collected to measure FPG, fasting insulin, blood lipids (including TC, TG, LDL-C, and HDL-C), DPP4 activity and active GLP-1. Blood samples were also drawn at 30 and 120 min after a 75 g glucose load to measure glucose and insulin concentrations. Demographic characteristics, life style risk factors, anthropometric parameters and venous blood samples were both collected or determined at baseline and four years later.

### Data collection

Plasma glucose levels were measured using a hexokinase enzymatic method. Insulin was measured by a radioimmunoassay with human insulin as a standard (Linco, St Charles, MO). TG, TC, LDL-C, and HDL-C levels were determined enzymatically. Plasma DPP4 activity was determined as the rate of cleavage of 7-amino-4- methylcoumarin (AMC) from the synthetic substrate H-glycyl-prolyl-AMC (H-Gly-Pro-AMC; Biovision, San Francisco, California, U.S.A.). It is expressed as the amount of cleaved AMC per minute per mL (nmol/min/mL). DPP4 activity was measured in the absence or the presence of sitagliptin, a specific DPP4 inhibitor, to test the specificity of the enzymatic assay. In our samples, sitagliptin inhibited the assayed activity by >95%. The intra-assay and inter-assay coefficients of variation were 2.13% and 8.56%, respectively. Samples for active GLP-1 were collected into iced Vacutainer tubes prepared with EDTA and DPP4 inhibitor for preventing degradation of active GLP into truncated, inactive GLP-1. Active GLP-1 which includes GLP-1(7-37) and GLP-1(7-36) was measured by enzyme-linked immunosorbent assay (Millipore, U.S.A.). The intra-assay and inter-assay coefficients of variation were 1.74% and 9.87%, respectively. Blood samples for measuring DPP4 activity and active GLP-1 levels were stored at −80 °C and subsequently DPP4 activity and active GLP-1 levels of all samples were measured within six months after the sample collection. The homeostasis model assessment of insulin resistance (HOMA-IR) was calculated from FPG and fasting insulin levels using the equation: FPG (mmol/L) × fasting insulin (μIU/ml)/22.5.

### Statistical analysis

All of the statistical analyses were performed using the SPSS 16.0 software (SPSS Inc., Chicago, IL, USA). Data were expressed as means ± standard deviation, median (interquartile range), or percentage for normally distributed continuous various, abnormally distributed continuous variables, and categorical variables, respectively. Abnormally distributed variables including fasting insulin, 2-hour insulin, HOMA-IR and TG were logarithmically transformed before analysis. We divided the study population into quintiles of plasma DPP4 activity with cut points 5.57, 6.05, 6.50, 7.00 for men and 5.11, 5.95, 6.39, 7.02 for women, and plasma active GLP-1 with cut points 2.55, 2.80, 3.14, 3.71 for men and 2.45, 2.80, 3.13, 3.82 for women respectively. We evaluated the association of baseline DPP4 activity and active GLP-1 with the incidence of new cases of metabolic syndrome and with the incidence of new cases of each component of the metabolic syndrome at the follow-up visit. To evaluate the incidence of new cases of each component, we excluded subjects with the presence of that specific component at baseline. Clinical and biochemical characteristics were compared by ANCOVA or χ2 tests. The DPP4 activity and active GLP-1′S predictive value for insulin resistance were quantified by multiple linear regressions. Logistic regression models were calculated to identify independent relations between DPP4 activity, active GLP-1 and incident metabolic syndrome. Five models were calculated: one crude model was adjusted for age, sex, BMI, and a fifth model was additionally adjusted for SBP, FPG, fasting insulin, TG, HDL-C, family history of diabetes, physical activity, smoking and alcohol consumption (fully adjusted model). Odds ratios (ORs) and 95% CIs are reported. To evaluate the added discrimination provided by DPP4 activity or active GLP-1 to predict incident cases of metabolic syndrome beyond the information provided by the components of the metabolic syndrome, we compared the areas under the receiver operating characteristic (ROC) curve in models that included BMI HDL cholesterol, TG, systolic blood pressure (SBP), fasting plasma glucose (FPG), and urine albumin-creatinine ratio(ACR) with and without DPP4 activity or active GLP-1.

## Results

During 4 years follow-up, 131men (15.2%) and 174 women (14.8%) developed metabolic syndrome. Baseline BMI, WHR, SBP, diastolic blood pressure (DBP), 2 h-PG, 2 h-insulin, TG, total cholesterol (TC), HOMA-IR were significantly higher and HDL cholesterol was significantly lower in men and women who developed metabolic syndrome compared with those who did not (Table 1).

**Table 1:** Baseline characteristics of study population by incident metabolic syndrome.

	Men			Women		
	No metabolic syndrome	Metabolic syndrome	P	No metabolic syndrome	Metabolic syndrome	P
N(%)	732(84.8)	131(15.2)	-	1005(85.2)	174(14.8)	-
Age(years)	43.6±14.6	48.8±13.5	0.000	42.4±13.4	50.2±13.4	0.000
Current smoking, n (%)	168(23.0)	41 (31.3)	0.040	51(5.1)	18(10.3)	0.006
Alcohol consumption, n (%)	328(44.8)	64(48.9)	0.640	141(14.0)	30(17.2)	0.267
Leisure-time physical activity, n (%)	358(48.9)	87(66.4)	0.000	661(65.8)	118(67.8)	0.599
Family history of diabetes, n (%)	111(15.2)	23(17.6)	0.486	157(15.6)	24(13.8)	0.537
BMI(kg/m^2^) ^a^	22.61±3.36	26.03±4.99	0.000	22.68±3.86	24.76±4.64	0.000
WHR^a^	0.86±0.08	0.92±0.06	0.000	0.83±0.08	0.91±0.10	0.000
SBP(mmHg) ^a^	114.1±14.9	128.0±21.4	0.000	112.4±16.7	126.9±25.0	0.000
DBP(mmHg) ^a^	74.8±9.7	83.2±12.0	0.000	74.0±10.3	81.9±13.9	0.000
FPG (mmol/L) ^a^	4.61±0.60	4.75±0.57	0.068	4.61±0.55	4.74±0.64	0.082
2 h-PG (mmol/L) ^a^	5.30±1.21	5.80±1.19	0.000	5.46±1.10	5.92±1.06	0.000
Fasting insulin((μIU/ml) ^a^	6.48(4.88,8.41)	8.00(5.45,10.08)	0.000	6.56(4.95,8.59)	7.30(5.46,9.52)	0.086
2 h-insulin(μIU/ml) ^a^	18.37(10.70,30.47)	27.72(15.82,46.28)	0.027	21.20(13.11,37.25)	25.78(13.22,50.23)	0.021
TG (mmol/L) ^a^	1.16(0.82,1.60)	2.07(1.57,2.87)	0.000	1.07(0.81,1.47)	1.77(1.27,2.32)	0.000
TC (mmol/L) ^a^	4.47±1.00	5.00±0.90	0.000	4.57±1.48	4.96±1.32	0.019
LDL-C (mmol/L) ^a^	2.65±0.78	2.96±0.76	0.001	2.65±0.76	2.85±0.98	0.203
HDL-C (mmol/L) ^a^	1.24±0.33	1.09±0.25	0.000	1.33±0.34	1.21±0.39	0.000
HOMA-IR^ a^	1.32(0.99, 1.69)	1.67(1.13,2.25)	0.000	1.32(0.99,1.80)	1.53(1.15,2.07)	0.032
Metabolic syndrome components, n (%)						
High blood pressure	85(11.6)	60(45.8)	0.000	110(10.9)	79(45.4)	0.000
High TG	157(21.4)	92(70.2)	0.000	164(16.3)	96(55.2)	0.000
Low HDL-C	79(10.8)	33(25.2)	0.000	139(13.8)	68(39.1)	0.000
Central obesity	180(24.6)	101(77.1)	0.000	360(35.8)	140(80.5)	0.000
Microalbuminuria	78(10.7)	42(32.1)	0.000	135(13.4)	69(39.7)	0.000

Data were expressed as means ± standard deviation, median (interquartile range), or percentage for normally distributed continuous various, abnormally distributed continuous variables, and categorical variables, respectively. Cigarette smoking was defined as having smoked at least 100 cigarettes in one's lifetime. Alcohol consumption was defined as consumption of ≥30 g of alcohol per week for 1 year or more. Regular leisure-time physical activity was defined as participation in ≥30 min of moderate or vigorous activity per day at least 3 days per week. ^a^ adjusted for age.

As per male and female, there was no significant statistical difference between the levels of DPP4 activity and active GLP-1 (as shown in Table S3 in File S1). Both in male and female, in comparison to the age group ≤30 years, age ≥61 years have increased level of DPP4 activity. Further, in comparison to the women between the age group 31–40 years, age group ≥51-years-old has significantly lower level of active GLP-1 (Figure S2 in File S1). DPP4 activity at baseline were significantly higher in subjects who developed metabolic syndrome compared with those who did not in both men and women whereas active GLP-1 levels was significantly lower (all P<0.001). A similar association was observed between DPP4 activity, active GLP-1 and each component of the metabolic syndrome except for high diastolic blood pressure and microalbuminuria (all P<0.05). Furthermore, plasma DPP4 activity progressively increased with the number of metabolic syndrome components developed by study participants over follow-up whereas active GLP-1 decreased progressively (P for trend <0.001 in both men and women) (Table 2).

**Table 2:** Baseline DPP4 activity and active GLP-1 according to presence or absence of components of new-onset metabolic syndrome.

	Men(n = 863)			Women(n = 1179)		
	Present	Absent	P	Present	Absent	P
DPP4 activity (nmol/min/ml)						
Metabolic syndrome	7.39±3.34	5.80±2.04	0.000	7.77±3.49	5.73±2.09	0.000
High SBP	6.91±2.96	5.95±2.26	0.001	7.20±3.41	5.90±2.29	0.000
High DBP	6.01±2.95	6.04±2.25	0.753	6.98±3.19	5.89±2.31	0.000
High TG	6.55±2.81	5.83±2.11	0.000	6.62±3.18	5.86±2.19	0.000
Low HDL-C	6.65±3.22	5.95±2.18	0.003	6.42±3.04	5.94±2.31	0.012
Central obesity	6.43±2.93	5.85±1.99	0.003	6.66±3.01	5.93±1.95	0.002
Microalbuminuria	6.01±2.66	6.04±2.30	0.785	7.99±1.55	6.11±1.33	0.000
No. of components						
0	5.82±1.92			5.70±1.87		
1	5.56±1.96			5.77±2.03		
2	6.45±2.24			5.98±2.47		
3	7.17±3.72			7.16±3.36		
> = 4	7.71±4.32			8.40±4.69		
P for trend	0.000			0.000		
Active GLP-1 (pmol/L)						
Metabolic syndrome	2.82±0.87	3.17±1.00	0.000	2.77±0.95	3.19±1.07	0.000
High SBP	2.85±0.99	3.14±0.99	0.022	2.80±1.04	3.14±1.07	0.000
High DBP	2.85±0.92	3.15±0.99	0.004	3.01±1.06	3.15±1.06	0.273
High TG	2.96±0.86	3.18±1.03	0.006	2.96±1.04	3.18±1.06	0.015
Low HDL-C	2.89±0.89	3.13±1.00	0.017	2.99±0.91	3.16±1.09	0.030
Central obesity	2.99±0.95	3.17±1.00	0.021	2.71±1.09	3.17±1.04	0.000
Microalbuminuria	3.06±1.09	3.12±0.97	0.625	3.05±1.05	3.15±1.06	0.284
No. of components						
0	3.24±1.01			3.21±1.03		
1	3.11±0.98			3.15±1.06		
2	3.16±1.01			3.17±1.14		
3	2.61±0.72			2.93±0.99		
> = 4	2.66±0.87			2.56±0.68		
P for trend	0.000			0.000		

Data were expressed as means ± standard deviation.

Four-year longitudinal studies showed that baseline DPP4 activity was an independent predictor of an increase in fasting insulin and HOMA-IR in both men and women after adjustment for age, BMI, SBP, TG, HDL-C(all P<0.01) (Table 3). After a follow-up of over 4 years, the proportions of subjects who developed new-onset metabolic syndrome, high blood glucose, high blood pressure, high TG, low HDL cholesterol, high WHR, and high urine ACR were 14.9, 19.5, 15.0, 24.1, 18.0, 20.1 and 15.9%, respectively. In multivariable -adjusted models [model 5 (Table 4 and Table 5)], the OR for developing metabolic syndrome comparing subjects in the highest with those in the lowest quintile of baseline DPP4 activity and active GLP-1 were 2.82, 0.45 for men and 2.48, 0.36 for women respectively. The corresponding ORs for high blood pressure, high TG, low HDL cholesterol, high WHR and high urine ACR according to baseline DPP4 activity were 3.66, 2.30, 2.84, 2.53 and 1.90, according to baseline active GLP-1 were 0.51, 0.19, 0.29, 0.38 and 1.10 respectively (Table S1-S2 in File S1).

**Table 3:** Standardized coefficients (β) from the multiple linear regression analysis of glucose metabolism in the 4-year longitudinal study.

	Change in insulin(μU/ml)		Change in glucose(mmol/L)		Change in HOMA-IR	
	β	p	β	p	β	p
All^a^						
DPP4 activity	0.113	0.000	0.105	0.000	0.131	0.000
Active GLP-1	−0.048	0.031	0.001	0.949	−0.030	0.184
Men^b^						
DPP4 activity	0.116	0.001	0.095	0.006	0.108	0.002
Active GLP-1	−0.062	0.071	−0.015	0.672	−0.033	0.337
Women^b^						
DPP4 activity	0.105	0.000	0.112	0.000	0.157	0.000
Active GLP-1	−0.041	0.166	0.013	0.647	−0.030	0.313

a Sex,age,SBP, BMI,TG,HDL-C were included in the regeression model.

b Age,SBP, BMI,TG,HDL-C were included in the regression model.

**Table 4:** ORs for new-onset metabolic syndrome in men according to baseline DPP4 activity and active GLP-1.

	Q 1	Q2	Q3	Q 4	Q5
DPP4 activity(nmol/ml/min)	≤5.57	5.58–6.05	6.06–6.50	6.51–7.00	>7.00
New-onset metabolic syndrome	13(7.6)	17(9.8)	29(16.1)	27(16.5)	45(26.0)
Metabolic syndrome					
Model 1	1	1.40(0.64–3.06)0.397	2.64(1.29–5.41)0.008	2.76(1.34–5.69)0.006	3.79(1.91–7.52)0.000
Model 2	1	1.27(0.58–2.78)0.558	2.58(1.26–5.29)0.009	2.76(1.33–5.70)0.006	3.40 (1.71–6.80)0.001
Model 3	1	1.31(0.59–2.89)0.508	2.62(1.28–5.36)0.009	2.73(1.32–5.64)0.007	3.04(1.51–6.13)0.002
Model 4	1	1.36(0.60–3.09)0.459	2.73(1.29–5.76)0.009	2.67(1.25–5.71)0.012	2.78(1.35–5.75)0.006
Model 5	1	1.36(0.60–3.10)0.463	2.80(1.32–5.93)0.007	2.73(1.27–5.84)0.010	2.82(1.36–5.85)0.005
Active GLP-1 (pmol/L)	≤2.55	2.56–2.80	2.81–3.14	3.15–3.71	≥3.72
New-onset metabolic syndrome	35(20.0)	27(14.9)	26(16.0)	29(16.5)	14(8.3)
Metabolic syndrome					
Model 1	1	0.84(0.47–1.50)0.551	0.94(0.52–1.70)0.849	1.00(0.56–1.78)0.999	0.37(0.18–0.73)0.004
Model 2	1	1.04(0.57–1.90)0.902	1.09(0.60–2.00)0.772	1.15(0.64–2.08)0.630	0.43(0.22–0.87)0.018
Model 3	1	1.12(0.61–2.05)0.723	1.16(0.63–2.13)0.637	1.21(0.67–2.16)0.534	0.42(0.21–0.86)0.017
Model 4	1	1.06(0.57–1.99)0.856	1.23(0.65–2.33)0.526	1.17(0.67–2.19)0.621	0.46(0.22–0.95)0.035
Model 5	1	1.03(0.55–1.94)0.924	1.30(0.68–2.48)0.427	1.23(0.66–2.29)0.517	0.45(0.22–0.94)0.034

Data are OR (95% CI) P or n (%).

Model 1 (adjusted for Age, BMI).

Model 2 (Model 1+ SBP).

Model 3 (Model 2 + FPG + Fasting insulin).

Model 4 (Model 3 + TG +HDL-C).

Model 5 (Model 4+ family history + physical activity + smoking + alcohol consumption).

**Table 5:** ORs for new-onset metabolic syndrome in women according to baseline DPP4 activity and active GLP-1.

	Q 1	Q2	Q3	Q 4	Q5
DPP4 activity(nmol/ml/min)	≤5.11	5.12–5.95	5.96–6.39	6.40–7.02	>7.02
New-onset metabolic syndrome	21(8.8)	24(10.2)	25(10.6)	45(19.1)	59(25.2)
Metabolic syndrome					
Model 1	1	1.26(0.67–2.34)0.476	1.26(0.68–2.34)0.466	2.54(1.45–4.45)0.001	3.21(1.86–5.52)0.000
Model 2	1	1.24(0.66–2.32)0.501	1.27(0.68–2.37)0.447	2.51(1.43–4.42)0.001	2.74(1.57–4.75)0.000
Model 3	1	1.25(0.67–2.34)0.487	1.28(0.69–2.38)0.435	2.55(1.45–4.48)0.001	2.71(1.56–4.71)0.000
Model 4	1	1.37(0.72–2.60)0.336	1.22(0.64–2.32)0.541	2.58(1.45–4.61)0.001	2.43(1.38–4.29)0.002
Model 5	1	1.40(0.74–2.66)0.306	1.27(0.66–2.42)0.474	2.67(1.49–4.78)0.001	2.48(1.40–4.39)0.002
Active GLP-1 (pmol/L)	≤2.45	2.46–2.80	2.81–3.13	3.14–3.82	≥3.83
New-onset metabolic syndrome	55(23.3)	36(14.8)	40(17.2)	23(9.9)	20(8.5)
Metabolic syndrome					
Model 1	1	0.56(0.35–0.91)0.018	0.69(0.43–1.09)0.114	0.37(0.22–0.63)0.000	0.34(0.19–0.59)0.000
Model 2	1	0.57(0.35–0.92)0.021	0.72(0.45–1.15)0.163	0.36(0.21–0.61)0.000	0.35(0.20–0.62)0.000
Model 3	1	0.56(0.35–0.91)0.019	0.70(0.44–1.12)0.138	0.35(0.20–0.60)0.000	0.35(0.20–0.62)0.000
Model 4	1	0.60(0.37–0.99)0.047	0.66(0.40–1.07)0.092	0.36(0.21–0.63)0.000	0.37(0.21–0.65)0.001
Model 5	1	0.62(0.37–1.01)0.057	0.66(0.40–1.08)0.100	0.36(0.20–0.63)0.000	0.36(0.20–0.64)0.001

Data are OR (95% CI) P or n (%).

Model 1 (adjusted for Age, BMI).

Model 2 (Model 1+ SBP).

Model 3 (Model 2 + FPG + Fasting insulin).

Model 4 (Model 3 + TG +HDL-C).

Model 5 (Model 4+ family history + physical activity + smoking + alcohol consumption).

We then evaluated how well baseline DPP4 activity and active GLP-1 levels predict incident metabolic syndrome beyond the information provided by baseline levels of metabolic syndrome components. The area under the ROC curve to predict incident metabolic syndrome using BMI HDL-C, TG, SBP, FPG and ACR was 0.783(95% CI 0.756–0.810). After DPP4 activity or active GLP-1 were added to the model, the corresponding areas under the ROC curve were 0.827(0.801–0.852) and 0.795(0.770–0.821) respectively. The P values for the comparison in areas under the ROC curve for the models with and without DPP4 activity or active GLP-1 levels were 0.021and 0.53(Figure S1 in File S1).

## Discussion

In this prospective study, we demonstrate, for the first time, that plasma DPP4 activity predict the onset of IR and metabolic syndrome. Plasma DPP4 activity is also a strong positive predictor of the total number of components of the metabolic syndrome developed and of each individual component of the metabolic syndrome

Lamers et al. [6]have proved that enlargement of visceral adipocytes and adipose tissue inflammation enhance the release of DPP4 from the fat cell to circulation, moreover, they found that circulating DPP4 concentrations correlated with various classic markers for the metabolic syndrome, namely, waist circumference, BMI, plasma triglycerides, HOMA-IR and fat cell volume. Kirino et al. [10] also reported that plasma DPP4 activity correlates with BMI in healthy young people. In our study, we found that plasma DPP4 activity was significantly higher in subjects who had higher WHR, blood pressure, blood lipid and HOMA-IR, furthermore, we also found that increased DPP4 activity is an independent predictor of metabolic syndrome and its components in our prospective study. Consequently, we speculated that in a relatively early stage, the various factors of insulin resistance or the components of the metabolic syndrome not only have a close relationship with DPP4 protein level, but they may also be closely related with DPP4 activity. Lamers et al. have documented that DPP4 induce insulin resistance in an autocrine and paracrine fashion at the level of Akt in three different primary cell types, namely, adipocytes, skeletal muscle, and smooth muscle cells, enzymatic activity of DPP4 seems to be involved in this process. In our study, we demonstrated for the first time that baseline plasma DPP4 activity was an independent predictor of an increase in insulin resistance in population-based prospective study. However, our study did not explore much about the mechanism by which increase in DPP4 activity lead to insulin resistance, that's why we are still not sure that DPP4 activity can lead to increase in insulin resistance.

We performed ROC curve analyses to evaluate the additional predictive ability of DPP4 activity beyond the information provided by the components of the metabolic syndrome at baseline. Within a model including BMI HDL-C, TG, SBP, FBG and ACR, DPP4 activity did significantly increase the area under the ROC curve, thereby demonstrating that in Chinese population, plasma DPP4 activity may increase the predictive ability for identification of subjects at risk for developing new-onset metabolic syndrome beyond that of the information provided by the components of the metabolic syndrome.

GLP-1, a member of the incretin hormone family, is found to be involved in insulin secretion and beta-cell proliferation in preclinical studies [11]–[12]. There are two forms of circulating active GLP-1 secreted after meal ingestion: GLP-1(7-37) and GLP-1(7-36), both peptides are equipotent and exhibit identical plasma half-lives and biological activities acting through the same receptor [13]. Since DPP4 is involved in the degradation of circulating active GLP-1 to biologically inactive fragments, plasma active GLP-1 level could also be associated with the development of insulin resistance and metabolic syndrome. In our study, we found that fasting active GLP-1 can not predict the development of insulin resistance and incident metabolic syndrome beyond the information provided by its components, although our multiple logistic regression analyses indicated that fasting active GLP-1 predict the onset of metabolic syndrome and some of its components. The specific reason for this inconsistency is still unknown, we speculate that this mismatch may be related to pattern of active GLP-1 secretion. Since plasma active GLP-1 level increase significantly after meal ingestion and since it is responsible for a large proportion of postprandial insulin secretion, we can not ignore the possibility that fasting active GLP-1 is not an effective predictor of the onset of insulin resistance and metabolic syndrome in apparently healthy Chinese, postprandial plasma active GLP-1 may play a more important role than fasting plasma active GLP-1 in predicting incident metabolic syndrome. Prospective studies are still needed to evaluate the predictive value of postprandial plasma active GLP-1 to identify individuals at high risk of new-onset metabolic syndrome.

Some limitations of our study should also be considered. Firstly, the follow-up period of our cohort was only 4 years, and we could not evaluate whether the association between DPP4 activity, active GLP-1 and incident metabolic syndrome would persist in longer follow up. Secondly, we did not evaluate the predictive value of postprandial plasma active GLP-1 to identify individuals at high risk of new-onset metabolic syndrome. Lastly, this study is an epidemiological study and somehow it fails to address the precise role of DPP4 and GLP1 in the pathogenesis of metabolic syndrome and insulin resistance which is needed to be elucidated by further basic investigation.

In summary, we have shown prospectively that increased fasting plasma DPP4 activity independently predict incident metabolic syndrome and insulin resistance in apparently healthy Chinese, and it may be considered as a novel marker of metabolic syndrome and insulin resistance. These findings have implications for increasing our understanding of the aetiology of metabolic syndrome and merit further study in future studies that help to clarify causality and advance this area of research.

## Supporting Information

File S1 Table S1. ORs for new-onset metabolic syndrome components according to baseline DPP4 activity (nmol/ml/min); Table S2. ORs for new-onset metabolic syndrome components according to baseline active GLP-1(pmol/L); Table S3. Comparison of DPP4 activity and active GLP-1 between men and women according to age; Figure S1. The area under the ROC curve to predict incident metabolic syndrome using Model1, Model2 and Model3; Figure S2. Sex-specific DPP4 activity and active GLP-1 levels according to age.
(DOC)
