# Supplementary material for: Increased Plasma DPP4 Activity Is an Independent Predictor of the Onset of Metabolic Syndrome in Chinese over 4 Years: Result from the China National Diabetes and Metabolic Disorders Study
Source: PLoS One. 2014 Mar 19;9(3):e92222. doi: 10.1371/journal.pone.0092222 (PMC3960228; doi:10.1371/journal.pone.0092222)

Table S1 -ORs for new-onset metabolic syndrome components according to baseline DPP4 activity (nmol/ml/min)

|  | Q 1 | | Q2 | | Q3 | | Q 4 | | Q 5 | |
| --- | --- | --- | --- | --- | --- | --- | --- | --- | --- | --- |
|  | ≤5.21 | | 5.22-6.00 | | 6.01-6.45 | | 6.46-7.02 | | ≥7.03 | |
|  | OR (95%) | P | OR (95%) | P | OR (95%) | P | OR (95%) | P | OR (95%) | P |
| High blood pressure |  |  |  |  |  |  |  |  |  |  |
| Model 1 | 1 | - | 0.96(0.57-1.62) | 0.866 | 1.78(1.11-2.85) | 0.016 | 1.61(1.00-2.60) | 0.050 | 3.94(2.53-6.12) | 0.000 |
| Model 2 | 1 | - | 0.96(0.57-1.62) | 0.872 | 1.78(1.11-2.85) | 0.016 | 1.61(1.00-2.59) | 0.051 | 3.88(2.49-6.05) | 0.000 |
| Model 3 | 1 | - | 0.97(0.57-1.64) | 0.898 | 1.79(1.12-2.87) | 0.015 | 1.61(1.00-2.60) | 0.050 | 3.84(2.46-6.00) | 0.000 |
| Model 4 | 1 | - | 0.98(0.58-1.66) | 0.931 | 1.82(1.13-2.93) | 0.014 | 1.59(0.98-2.57) | 0.060 | 3.73(2.38-5.84) | 0.000 |
| Model 5 | 1 | - | 0.94(0.55-1.60) | 0.821 | 1.76(1.09-2.85) | 0.020 | 1.56(0.96-2.53) | 0.073 | 3.66(2.33-5.75) | 0.000 |
| High TG |  |  |  |  |  |  |  |  |  |  |
| Model 1 | 1 | - | 0.72(0.49-1.07) | 0.107 | 1.42(0.98-2.04) | 0.061 | 1.45(1.01-2.09) | 0.047 | 2.27(1.57-3.28) | 0.000 |
| Model 2 | 1 | - | 0.72(0.49-1.07) | 0.105 | 1.42(0.99-2.05) | 0.059 | 1.45(1.01-2.09) | 0.047 | 2.25(1.55-3.25) | 0.000 |
| Model 3 | 1 | - | 0.72(0.49-1.08) | 0.110 | 1.42(0.99-2.05) | 0.059 | 1.45(1.01-2.09) | 0.046 | 2.23(1.54-3.23) | 0.000 |
| Model 4 | 1 | - | 0.71(0.48-1.06) | 0.090 | 1.41(0.98-2.03) | 0.067 | 1.46(1.02-2.11) | 0.041 | 2.28(1.57-3.31) | 0.000 |
| Model 5 | 1 | - | 0.72(0.48-1.07) | 0.099 | 1.42(0.99-2.06) | 0.060 | 1.48(1.03-2.13) | 0.037 | 2.30(1.58-3.34) | 0.000 |
| Low HDL cholesterol |  |  |  |  |  |  |  |  |  |  |
| Model 1 | 1 | - | 1.30(0.83-2.02) | 0.254 | 1.32(0.84-2.06) | 0.226 | 2.01(1.32-3.06) | 0.001 | 3.17(2.11-4.77) | 0.000 |
| Model 2 | 1 | - | 1.25(0.80-1.95) | 0.332 | 1.32(0.85-2.07) | 0.219 | 2.03(1.33-3.10) | 0.001 | 2.91(1.92-4.39) | 0.000 |
| Model 3 | 1 | - | 1.25(0.80-1.96) | 0.327 | 1.32(0.85-2.07) | 0.221 | 2.03(1.33-3.10) | 0.001 | 2.93(1.94-4.44) | 0.000 |
| Model 4 | 1 | - | 1.30(0.83-2.04) | 0.252 | 1.35(0.86-2.12) | 0.197 | 2.07(1.35-3.16) | 0.001 | 2.82(1.85-4.29) | 0.000 |
| Model 5 | 1 | - | 1.30(0.83-2.05) | 0.255 | 1.36(0.87-2.15) | 0.181 | 2.08(1.36-3.19) | 0.001 | 2.84(1.86-4.33) | 0.000 |
| High WHR |  |  |  |  |  |  |  |  |  |  |
| Model 1 | 1 | - | 1.11(0.67-1.84) | 0.684 | 2.53(1.58-4.06) | 0.000 | 1.77(1.10-2.85) | 0.019 | 2.78(1.74-4.46) | 0.000 |
| Model 2 | 1 | - | 1.10(0.66-1.83) | 0.715 | 2.62(1.63-4.21) | 0.000 | 1.83(1.13-2.95) | 0.014 | 2.69(1.67-4.32) | 0.000 |
| Model 3 | 1 | - | 1.10(0.66-1.84) | 0.703 | 2.62(1.63-4.21) | 0.000 | 1.83(1.13-2.95) | 0.013 | 2.64(1.63-4.25) | 0.000 |
| Model 4 | 1 | - | 1.14(0.69-1.91) | 0.607 | 2.68(1.66-4.34) | 0.000 | 1.83(1.12-2.96) | 0.015 | 2.53(1.56-4.10) | 0.000 |
| Model 5 | 1 | - | 1.14(0.68-1.91) | 0.613 | 2.66(1.64-4.31) | 0.000 | 1.82(1.12-2.96) | 0.016 | 2.53(1.55-4.11) | 0.000 |
| High ACR |  |  |  |  |  |  |  |  |  |  |
| Model 1 | 1 | - | 0.70(0.43-1.16) | 0.167 | 1.62(1.06-2.50) | 0.027 | 1.53(1.00-2.36) | 0.051 | 2.15(1.43-3.25) | 0.000 |
| Model 2 | 1 | - | 0.70(0.42-1.14) | 0.151 | 1.62(1.05-2.49) | 0.028 | 1.53(1.00-2.35) | 0.053 | 2.02(1.33-3.06) | 0.001 |
| Model 3 | 1 | - | 0.71(0.43-1.16) | 0.172 | 1.63(1.06-2.50) | 0.027 | 1.53(1.00-2.36) | 0.052 | 1.96(1.29-2.98) | 0.002 |
| Model 4 | 1 | - | 0.73(0.44-1.20) | 0.212 | 1.64(1.06-2.52) | 0.026 | 1.52(0.98-2.34) | 0.059 | 1.88(1.23-2.88) | 0.003 |
| Model 5 | 1 | - | 0.73(0.44-1.20) | 0.213 | 1.64(1.06-2.53) | 0.027 | 1.52(0.99-2.34) | 0.059 | 1.90(1.24-2.90) | 0.003 |

Data are OR (95% CI) P or n (%).

Model 1 (adjusted for Age, sex, BMI)

Model 2 (Model 1+ SBP)

Model 3 (Model 2 + FPG + Fasting insulin)

Model 4 (Model 3 + TG +HDL-C)

Model 5 (Model 4+ family history + physical activity + smoking + alcohol consumption)

Table S2 -ORs for new-onset metabolic syndrome components according to baseline active GLP-1(pmol/L)

|  | Q1 | | Q2 | | Q3 | | Q4 | | Q5 | |
| --- | --- | --- | --- | --- | --- | --- | --- | --- | --- | --- |
|  | ≤2.50 | | 2.51-2.80 | | 2.81-3.14 | | 3.15-3.74 | | ≥3.75 | |
|  | OR (95%) | P | OR (95%) | P | OR (95%) | P | OR (95%) | P | OR (95%) | P |
| High blood pressure |  |  |  |  |  |  |  |  |  |  |
| Model 1 | 1 | - | 0.56(0.37-0.85) | 0.006 | 0.76(0.51-1.14) | 0.182 | 0.62(0.41-0.94) | 0.025 | 0.47(0.30-0.73) | 0.001 |
| Model 2 | 1 | - | 0.56(0.37-0.85) | 0.007 | 0.76(0.51-1.13) | 0.179 | 0.61(0.40-0.94) | 0.023 | 0.47(0.31-0.73) | 0.001 |
| Model 3 | 1 | - | 0.56(0.37-0.86) | 0.007 | 0.76(0.51-1.14) | 0.188 | 0.62(0.40-0.94) | 0.024 | 0.47(0.31-0.73) | 0.001 |
| Model 4 | 1 | - | 0.56(0.36-0.86) | 0.007 | 0.76(0.51-1.14) | 0.189 | 0.62(0.40-0.95) | 0.027 | 0.50(0.32-0.77) | 0.002 |
| Model 5 | 1 | - | 0.57(0.37-0.87) | 0.009 | 0.78(0.52-1.17) | 0.230 | 0.63(0.41-0.97) | 0.035 | 0.51(0.33-0.80) | 0.003 |
| High TG |  |  |  |  |  |  |  |  |  |  |
| Model 1 | 1 | - | 1.73(1.23-2.41) | 0.083 | 1.39(1.00-1.95) | 0.051 | 0.21(0.13-0.33) | 0.000 | 0.18(0.14-0.25) | 0.000 |
| Model 2 | 1 | - | 1.77(1.26-2.50) | 0.080 | 1.41(1.00-1.97) | 0.050 | 0.21(0.13-0.34) | 0.000 | 0.18(0.14-0.25) | 0.000 |
| Model 3 | 1 | - | 1.81(1.29-2.53) | 0.082 | 1.40(1.00-1.96) | 0.051 | 0.21(0.14-0.34) | 0.000 | 0.18(0.14-0.26) | 0.000 |
| Model 4 | 1 | - | 1.80(1.27-2.52) | 0.089 | 1.39(1.00-1.96) | 0.054 | 0.21(0.14-0.34) | 0.000 | 0.18(0.13-0.26) | 0.000 |
| Model 5 | 1 | - | 1.80(1.28-2.53) | 0.095 | 1.39(0.99-1.95) | 0.061 | 0.22(0.14-0.35) | 0.000 | 0.19(0.14-0.28) | 0.000 |
| Low HDL cholesterol |  |  |  |  |  |  |  |  |  |  |
| Model 1 | 1 | - | 0.72(0.51-1.01) | 0.059 | 0.70(0.50-1.00) | 0.048 | 0.45(0.31-0.66) | 0.000 | 0.26(0.14-0.45) | 0.000 |
| Model 2 | 1 | - | 0.76(0.54-1.07) | 0.119 | 0.74(0.52-1.05) | 0.089 | 0.46(0.32-0.68) | 0.000 | 0.27(0.15-0.46) | 0.000 |
| Model 3 | 1 | - | 0.76(0.54-1.07) | 0.120 | 0.73(0.52-1.05) | 0.087 | 0.46(0.32-0.68) | 0.000 | 0.27(0.15-0.46) | 0.000 |
| Model 4 | 1 | - | 0.78(0.55-1.10) | 0.159 | 0.76(0.53-1.08) | 0.126 | 0.46(0.32-0.68) | 0.000 | 0.28(0.15-0.48) | 0.000 |
| Model 5 | 1 | - | 0.80(0.56-1.14) | 0.220 | 0.75(0.52-1.08) | 0.121 | 0.44(0.30-0.65) | 0.000 | 0.29 (0.16-0.50) | 0.000 |
| High WHR |  |  |  |  |  |  |  |  |  |  |
| Model 1 | 1 | - | 0.69(0.47-1.03) | 0.067 | 0.72(0.48-1.07) | 0.102 | 0.49(0.32-0.74) | 0.001 | 0.36(0.13-0.62) | 0.000 |
| Model 2 | 1 | - | 0.73(0.49-1.09) | 0.122 | 0.75(0.50-1.12) | 0.154 | 0.50(0.33-0.76) | 0.001 | 0.36(0.13-0.62) | 0.000 |
| Model 3 | 1 | - | 0.73(0.49-1.09) | 0.122 | 0.74(0.49-1.10) | 0.138 | 0.50(0.33-0.76) | 0.001 | 0.36(0.14-0.61) | 0.000 |
| Model 4 | 1 | - | 0.72(0.48-1.08) | 0.109 | 0.76(0.50-1.15) | 0.189 | 0.51(0.33-0.78) | 0.002 | 0.37(0.14-0.62) | 0.000 |
| Model 5 | 1 | - | 0.72(0.48-1.08) | 0.114 | 0.75(0.50-1.13) | 0.173 | 0.49(0.32-0.76) | 0.001 | 0.38(0.13-0.64) | 0.000 |
| High ACR |  |  |  |  |  |  |  |  |  |  |
| Model 1 | 1 | - | 0.96(0.61-1.47) | 0.859 | 1.00(0.72-1.38) | 0.895 | 1.21(0.91-1.61) | 0.254 | 1.07(0.73-1.56) | 0.584 |
| Model 2 | 1 | - | 0.96(0.61-1.47) | 0.805 | 1.00(0.72-1.39) | 0.941 | 1.16(0.87-1.57) | 0.301 | 1.08(0.73-1.57) | 0.573 |
| Model 3 | 1 | - | 0.97(0.62-1.52) | 0.845 | 1.01(0.73-1.40) | 0.901 | 1.15(0.86-1.55) | 0.384 | 1.07(0.73-1.56) | 0.540 |
| Model 4 | 1 | - | 0.98(0.62-1.53) | 0.840 | 0.95(0.65-1.32) | 0.721 | 1.16(0.87-1.57) | 0.305 | 1.10(0.75-1.61) | 0.421 |
| Model 5 | 1 | - | 0.97(0.62-1.52) | 0.835 | 0.94(0.65-1.30) | 0.698 | 1.14(0.84-1.53) | 0.455 | 1.10(0.75-1.60) | 0.412 |

Data are OR (95% CI) P or n (%).

Model 1 (adjusted for Age, sex, BMI)

Model 2 (Model 1+ SBP)

Model 3 (Model 2 + FPG + Fasting insulin)

Model 4 (Model 3 + TG +HDL-C)

Model 5 (Model 4+ family history + physical activity + smoking + alcohol consumption)

Table S3 -Comparison of DPP4 activity and active GLP-1 between men and women according to age

|  | Men(n=863) | Women(n=1179) | P |
| --- | --- | --- | --- |
| DPP4 activity  (nmol/min/ml) |  |  |  |
| ≤30 | 5.74±2.50(198) | 5.92±1.83(243) | NS |
| 31-40 | 5.97±1.98(182) | 5.73±2.32(290) | NS |
| 41-50 | 6.05±2.11(151) | 6.01±2.53(262) | NS |
| 51-60 | 6.20±2.42(186) | 6.08±2.82(220) | NS |
| ≥61 | 6.32±2.67(146) | 6.67±2.76(164) | NS |
| Total | 6.04±2.35(863) | 6.03±2.46(1179) | NS |
| Active GLP-1  (pmol/L) |  |  |  |
| ≤30 | 3.14±0.92(198) | 3.19±1.02(243) | NS |
| 31-40 | 3.18±1.13(182) | 3.24±1.11(290) | NS |
| 41-50 | 3.16±0.88(151) | 3.12±1.03(262) | NS |
| 51-60 | 3.06±0.99(186) | 3.03±1.13(220) | NS |
| ≥61 | 3.01±1.00(146) | 3.00±0.96(164) | NS |
| Total | 3.11±0.99(863) | 3.13±1.06(1179) | NS |

Data are expressed as means ± standard deviation.

Figure S1: The area under the ROC curve to predict incident metabolic syndrome using Model1, Model2 and Model3


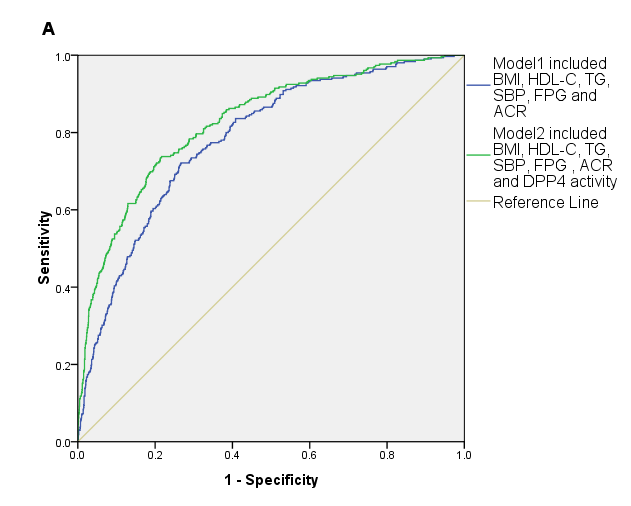

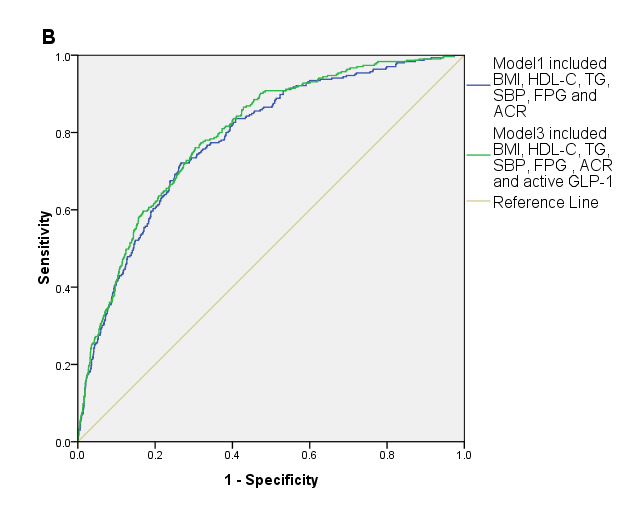


Model1 included BMI, HDL-C, TG, SBP, FPG and ACR

Model2 included BMI, HDL-C, TG, SBP, FPG , ACR and DPP4 activity

Model3 included BMI, HDL-C, TG, SBP, FPG , ACR and active GLP-1

Figure S2：Sex-specific DPP4 activity and active GLP-1 levels according to age.


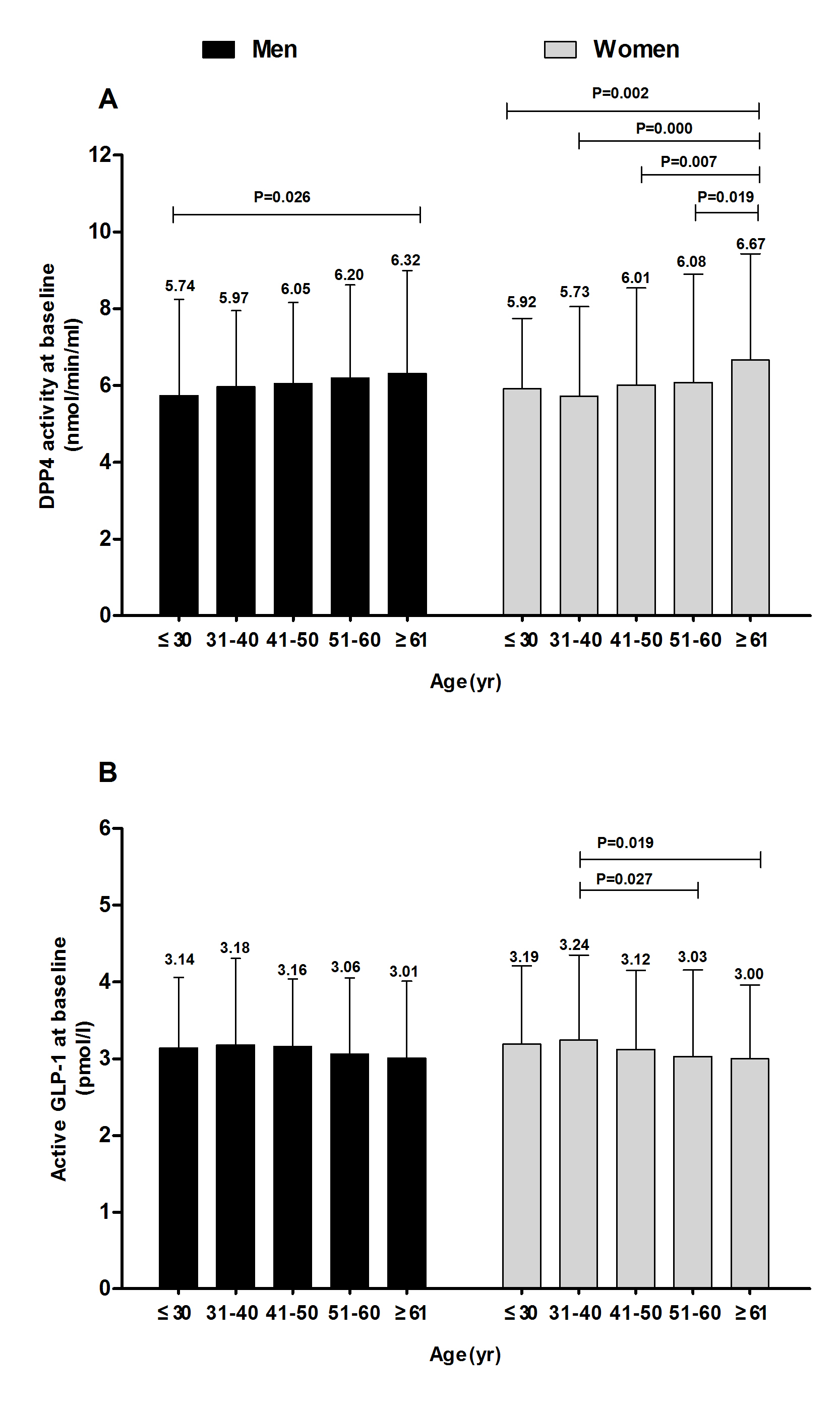

Supplement: File S1 — Table S1. ORs for new-onset metabolic syndrome components according to baseline DPP4 activity (nmol/ml/min); Table S2. ORs for new-onset metabolic syndrome components according to baseline active GLP-1(pmol/L); Table S3. Comparison of DPP4 activity and active GLP-1 between men and women according to age; Figure S1. The area under the ROC curve to predict incident metabolic syndrome using Model1, Model2 and Model3; Figure S2. Sex-specific DPP4 activity and active GLP-1 levels according to age. (DOC) [file pone.0092222.s001.doc]
